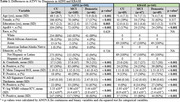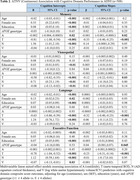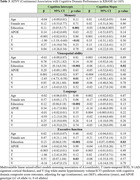# The Relative Contribution of Amyloid, Tau, Neurodegeneration, and Vascular Disease to Cognition and Cognitive Decline: A Cross‐National Study

**DOI:** 10.1002/alz70856_106457

**Published:** 2026-01-08

**Authors:** Jeremy A. Tanner, Diefei Chen, Min Soo Byun, Ileana De Anda‐Duran, Kacie D Deters, Martine Elbejjani, Evan Fletcher, A. Zarina Kraal, Dong Young Lee, Silvia Mejia‐Arango, Stefanie D Pina‐Escudero, Kwangsik Nho, Talia L. Robinson, C. Elizabeth Shaaban, Adam M. Staffaroni, Meichen Yu, Andrew J. Saykin, Paul K Crane, Dahyun Yi, Shannon L Risacher, ADNI, KBASE

**Affiliations:** ^1^ University of Texas Health San Antonio, San Antonio, TX, USA; ^2^ Department of Epidemiology, Bloomberg School of Public Health, Johns Hopkins University, Baltimore, MD, USA; ^3^ Seoul National University Medical Research Center, Seoul, Korea, Republic of (South); ^4^ Celia Scott Weatherhead Tulane University School of Public Health and Tropical Medicine, New Orleans, LA, USA; ^5^ University of California, Los Angeles Integrative Biology and Physiology (IBP), Los Angeles, CA, USA; ^6^ American University of Beirut, Beirut, Lebanon; ^7^ University of California, Davis, Davis, CA, USA; ^8^ Columbia University Irving Medical Center, New York, NY, USA; ^9^ The University of Texas Rio Grande Valley School of Medicine, Harlingen, TX, USA; ^10^ Memory and Aging Center, UCSF Weill Institute for Neurosciences, University of California, San Francisco, San Francisco, CA, USA; ^11^ Indiana University School of Medicine, Indianapolis, IN, USA; ^12^ Massachusetts General Hospital, Boston, MA, USA; ^13^ University of Pittsburgh, Pittsburgh, PA, USA; ^14^ Indiana University School of Medicine, Department of Radiology and Imaging Sciences, Indianapolis, IN, USA; ^15^ University of Washington, Seattle, WA, USA

## Abstract

**Background:**

Recent *in vivo* biomarker advancements have led to biological diagnostic and staging criteria for AD based on amyloid(“A”), tau(“T”), and neurodegeneration(“N”). However, the contribution of comorbid vascular(“V”) pathology and the relative influence of ATNV on cognitive performance and decline remains unclear, though is necessary to guide personalized diagnosis, prognosis, and care. We assessed the relative contribution of neuroimaging‐based ATNV measures to cognition in cross‐national studies.

**Method:**

Amyloid PET, tau PET, MRI, cognitive testing, and clinical evaluations were obtained in two prospective cohort studies with similar designs: ADNI3 in the US (*n* = 508; mean age=71±7, female=55%, education(yrs)=16.5±2.3) and KBASE in South Korea (*n* = 165; age=73±8, female=64%, education(yrs)=11.0±4.6). Continuous ATNV predictors included A=cortical centiloids, T=meta‐temporal SUVR, *N* = AD‐signature cortical thickness, and V=white matter hyperintensity volume. Clinical diagnoses of cognitively unimpaired (CU), mild cognitive impairment (MCI), and dementia were determined by clinical consensus. Cognitive testing was obtained annually with up to 4 years of follow‐up, and factor scores in each cognitive domain were used as outcomes. Parallel cross‐sectional analyses were performed within each cohort. ANOVA was performed to compare ATNV across clinical diagnoses. Multivariable linear mixed‐effect models were used to assess the association of baseline ATNV predictors with baseline and longitudinal cognitive outcomes adjusting for age, sex, education, and *APOE* status.

**Result:**

A, T, N, and V as continuous (mean) and binary (frequency of positivity) variables increased with clinical disease severity in both ADNI and KBASE (Table 1). In ADNI, T and N were associated with lower intercepts in each cognitive domain, V was associated with visuospatial and executive function intercepts, and A with the memory intercept (Table 2). Higher baseline T was associated with decline in each domain, and A was associated with memory decline. In KBASE, T was associated with lower memory and visuospatial intercepts, and N with visuospatial and executive function intercepts (Table 3). No factors (ATNV) were associated with cognitive decline over time in KBASE.

**Conclusion:**

Among ATNV measures, T and N are most strongly associated with cognition in individuals in the US and South Korea. T is most predictive of future cognitive decline in individuals in the US, though not replicated in Korea.